# COVID-19-Related Daily Stress Processes in College-Aged Adults: Examining the Role of Depressive Symptom Severity

**DOI:** 10.3389/fpsyg.2021.693396

**Published:** 2021-09-13

**Authors:** Jody L. Greaney, Ashley M. Darling, Jennifer R. Turner, Erika F. H. Saunders, David M. Almeida, Jacqueline Mogle

**Affiliations:** ^1^Department of Kinesiology, The University of Texas at Arlington, Arlington, TX, United States; ^2^Edna Bennett Pierce Prevention Research Center, The Pennsylvania State University, University Park, PA, United States; ^3^Department of Psychiatry, Penn State College of Medicine, Hershey, PA, United States; ^4^Department of Human Development and Family Studies, The Pennsylvania State University, University Park, PA, United States; ^5^Center for Healthy Aging, The Pennsylvania State University, University Park, PA, United States

**Keywords:** depression, daily stress, negative affect, mood, COVID-19 pandemic

## Abstract

Exposure to daily stressors specific to the COVID-19 pandemic (e.g., threat of infection) is associated with emotional distress, heightened stress reactivity, and increased depressive symptomology. Herein, we examined whether current depressive symptomology modulates the association between COVID-19-related daily stressor exposure and negative affective reactivity in young, otherwise healthy, college-aged adults. Fifty-eight adults (21 men; 22±3years) completed a daily web-based interview for eight consecutive days to assess COVID-19-related daily stress exposure and emotional responsiveness (September–November 2020). Depressive symptom severity was assessed using the Patient Health Questionnaire-9 (PHQ-9), and a score of ≥10 (range: 0–27) was used to define adults with a depressive episode (*n*=20). Participants reported at least one COVID-19-related stressor on 35.8% of interview days. Depressive symptomology did not predict the likelihood of exposure to a COVID-19-related stressor (*p*=0.46; OR=1.52; 95% CI: 0.492–4.718). However, negative affect (NA) was greater on days with an exposure to any COVID-19-specific daily stressor in adults with moderate-to-severe depressive symptoms (*b*=0.28, SE=0.093, *p*=0.003) but not in those without (*b*=0.009, SE=0.074, *p*=0.90), such that negative affective reactivity to COVID-19-related stressors was amplified in adults with a current depressive episode (*p*=0.019). Depressive symptomology did not moderate positive affective reactivity (*p*=0.686). Taken together, these data suggest that exposure to daily stressors related to COVID-19 further worsens NA in adults with a current depressive episode, potentially rendering them more susceptible to adverse mental health outcomes during the pandemic.

## Introduction

The global COVID-19 pandemic has profoundly impacted nearly all aspects of daily life. Beyond the direct threat of infection for physical health, the implementation of numerous lifestyle measures to slow disease spread (e.g., quarantine, lockdown, physical distancing, etc.) has had far-reaching psychological, social, and economic effects (Nicola et al., 2020; Pan et al., 2021). Although, the global pandemic is a novel “once-in-a-generation” *chronic* stressor, the unprecedented curbs on social interaction and the ensuing social isolation are significant sources of *daily* stress unique to the circumstances surrounding COVID-19 (Brooks et al., 2020; Taylor et al., 2020). These naturalistic events or hassles that arise from day-to-day living during the pandemic, examples of which include fears of infection and survival, financial insecurity, resource scarcity, and tension, boredom, and frustration among families in lockdown together, activate stress-responsive neurocircuitry and thereby have immediate consequences for psychological and physiological function (Almeida et al., 2009; Stawski et al., 2013; Greaney et al., 2019).

There is marked heterogeneity in the subjective appraisal of daily stressors (Stawski et al., 2008, 2013), and mounting evidence suggests that emotional responsiveness to daily stressors is even more predictive of long-term disease risk than daily stressor exposure by and of itself (Sin et al., 2016; Chiang et al., 2018; Leger et al., 2018). Importantly, chronic life stress (e.g., living through a pandemic) necessarily also contextualizes and influences the processing of everyday daily stress, contributing to heightened stress-related negative affective reactivity (Stawski et al., 2008; Sliwinski et al., 2009). To probe this link for COVID-19, previous investigators conducted a 28-day daily diary study assessing stress and emotions in community-dwelling adults (age range: 26–89years) immediately following government lockdown orders in April 2020 (Nelson and Bergeman, 2020). Greater daily worry related to the pandemic exacerbated affective reactivity to daily stressors, an effect that was more pronounced in young compared to older adults (Nelson and Bergeman, 2020). These data suggest that young college-aged adults may be particularly vulnerable to increased daily stress amidst the COVID-19 pandemic (McGinty et al., 2020; Nelson and Bergeman, 2020; Nwachukwu et al., 2020; Klaiber et al., 2021), perhaps owing to their stronger need for social interaction, employment uncertainty, and/or the additional stressors related to online learning, including unstable internet connectivity, additional financial burdens, and difficulty focusing.

In addition to the striking increases in the prevalence of mental health illness during the COVID-19 pandemic (Holmes et al., 2020; Pierce et al., 2020; Vindegaard and Benros, 2020), it appears that the psychological and emotional sequela of pandemic-related stress may be even further amplified in adults with underlying psychiatric comorbidities, particularly depressive disorders (Yao et al., 2020; Pan et al., 2021). Indeed, increases in pandemic-related stressors predicted increases in emotional distress and depressive symptomology, even when controlling for baseline depression (Duan et al., 2020; Shanahan et al., 2020; Zheng et al., 2021). In response to daily stress exposure, young otherwise healthy adults with major depressive disorder report greater increases in negative emotions (Bylsma et al., 2011; Booij et al., 2018). However, whether depressive symptomology similarly modulates negative affective reactivity to the unique daily stressors specific to COVID-19 in young college-aged adults remains unclear.

Given the long-term physiological and psychological consequences of increased exposure and negative affective reactivity to daily stressors (Sin et al., 2016; Chiang et al., 2018; Leger et al., 2018) and, separately, depression (Cuijpers and Smit, 2002; Lett et al., 2004; Whiteford et al., 2013; Gilsanz et al., 2015), a better understanding of the inter-relations between COVID-19-related daily stress processes and depressive symptom severity may provide insight into effective strategies to prevent or mitigate untoward health outcomes stemming from the pandemic. Because policies related to containment of the COVID-19 virus severely limit stress mitigation approaches that rely on group gatherings and increased community support to promote resilience, this line of inquiry has clear public health relevance. Therefore, as a necessary first step, the aim of this small pilot study was to examine exposure and affective reactivity to daily stressors during the pandemic in college-aged adults with a broad range of depressive symptom severity. We tested the novel hypothesis that negative affective reactivity [i.e., the association between COVID-19-related daily stress exposure and negative affect (NA)] would be stronger in adults with moderate-to-severe depressive symptoms compared to those without.

## Materials and Methods

All experimental procedures and protocols were approved by the Institutional Review Board at the University of Texas at Arlington (2020-0912). The investigation was conducted in accordance with the Declaration of Helsinki. The nature, risks, and benefits of all study procedures were explained to participants, and their verbal informed consent was obtained voluntarily prior to participation. Due to the ongoing pandemic-related restrictions to in-person research, contact with participants was limited to online participation.

### Participants

College-aged adults were recruited from The University of Texas at Arlington and surrounding community using common means of study advertisement (e.g., posting recruitment fliers, social media, etc.), and a total of 64 were enrolled. Thereafter, participants completed a web-based version of the Daily Inventory of Stressful Events (DISE) interview, adapted to include stressors related to COVID-19, for eight consecutive days, as is standard for this type of study (Almeida et al., 2009; Klaiber et al., 2021). Participants received text message and email reminders every evening at 5pm local time with the link to DISE interview. Of those enrolled, 58 participants (91%) completed at least one daily diary. In total, participants completed 7.6±1.1 diaries, with most completing all eight (*n*=47; 81%) and only two participants completing fewer than 6days. Data were collected from September 8, 2020 until November 11, 2020, with some participants completing the study before others.

### Assessment of Daily Stress

The DISE interview assesses multiple components of daily stressor exposure using stem questions, followed by open-ended probes, asking whether any of seven types of naturally-occurring stressors occurred in the previous 24h: argument, argument avoidance, stressful event at work or school, stressful event at home, stressful event related to racial/ethnic/sexual discrimination, network stress (i.e., stressful even that happened to a close friend or relative), or any other stressful event (Almeida et al., 2002). The DISE captures specific events that occurred the immediate 24h preceding each daily assessment and, therefore, is focused on the types of daily hassles that result from everyday life (as listed above), as well as experiences that may stem from chronic stress occurring over much longer durations (e.g., living in poverty, being in an abusive relationship, etc.). Obtaining daily stressor information over this short-time frame helps in alleviating concerns regarding ecological validity and retrospective memory distortions that can occur over longer periods of time (Almeida et al., 2002). A dichotomous variable was created to indicate the occurrence of any stressor that day (1=*yes*, 0=*no*) and the total number of stressors was calculated for each day.

### Assessment of COVID-19-Related Daily Stress

The DISE was expanded to also include daily stressors specifically related to the circumstances surrounding the COVID-19 pandemic. Items from other publicly available surveys on the impacts of COVID-19 were adapted for daily administration and responding (Nelson and Bergeman, 2020; Klaiber et al., 2021). These items asked whether participants experienced any of the following COVID-19-related daily stressors: financial problems, unable to spend time with others, challenges at home, trouble obtaining supplies, distressing news reports, experience of physical symptoms of COVID-19, difficulty completing work or school requirements, and greater work or home responsibilities compared to before the COVID-19 pandemic. As above, a dichotomous variable was created to indicate the occurrence of any COVID-19-related stressor that day (1=*yes*, 0=*no*) and the total number of COVID-19-related stressors was calculated for each day.

### Assessment of Positive and Negative Affect

To assess daily affect, participants rated the frequency of 13 positive (in good spirits, cheerful, extremely happy, calm and peaceful, satisfied, full of life, close to others, like you belong, enthusiastic, attentive, proud, active, and confident) and 14 negative (restless or fidgety, nervous, worthless, so sad nothing could cheer you up, everything was an effort, hopeless, lonely, afraid, jittery, irritable, ashamed, upset, angry, and frustrated) emotions using a five-point scale (0=*none of the time*, 1=*a little of the time*, 2=*some of the time*, 3=*most of the time*, and 4=*all of the time*; Mroczek and Kolarz, 1998; Kessler et al., 2002). Reliability for the positive affect items was 0.97 and for the negative affect items was 0.93. The emotion item ratings were averaged to obtain daily positive and negative affect scores and scores were aggregated for the eight interview days. Daily positive affect was 1.89±0.93 (range 0–4) and daily negative affect was 0.63±0.49 (range 0–3.5).

### Assessment of Depressive Symptom Severity

On the first day of the DISE interview, depressive symptom severity was assessed using the Patient Health Questionnaire-9 (PHQ-9), which provides a valid and sensitive index of symptomology based on the diagnostic criteria for DSM-5 depressive disorders (Spitzer et al., 1999; Kroenke et al., 2001). The PHQ-9 rates the frequency of the nine clinical symptoms of depression in the past 7days on a four-point Likert scale (0=*not at all*, 1=*several days*, 2=*more than half of the days*, and 3=*nearly every day*). Response options are used to calculate a total score (maximum=27) and symptom severity is quantified as none (0–4), mild (5–9), moderate (10–14), moderately severe (15–19), or severe (20–27). In addition to grading depressive symptom severity, the PHQ-9 can also be used to establish a depressive episode diagnosis (Kroenke et al., 2001). Because the present data were collected entirely remotely without participants visiting the laboratory, the presence of depressive symptoms of at least moderate severity (i.e., a PHQ-9 score of ≥10) was used to define the group of adults with a depressive episode. A PHQ-9 score of ≥10 has a sensitivity of 88% and a specificity of 88% for a diagnosis of major depression (Kroenke et al., 2001). The group of non-depressed adults had PHQ-9 score of <10. Importantly, PHQ-9 scores <10 very seldomly occur in major depression (Kroenke et al., 2001). These cut-off values were selected because, in the absence of a diagnostic interview or clinical diagnosis, symptom severity scores in the moderate to moderate-to-severe range are more likely to be indicative of a depressive episode (Spitzer et al., 1999; Kroenke et al., 2001, 2010).

### Data Analytical and Statistical Approach

Data were examined in a series of steps. First, we computed all summary scores and calculated descriptive statistics for all participants and days. We then examined descriptive statistics to determine the frequency of COVID-19-related stressors, other daily stressful events, and their daily co-occurrence. Multilevel modeling was used for all analyses, as is appropriate when data are nested as with the current daily diaries (days at level 1 nested in persons at level 2) and allows for the estimation of within-person relations among the variables of interest, as well as how person-level variables (e.g., depression symptom severity) modify those relations (Hox et al., 2017). In the current study, we first examined whether a depressive disorder predicted the likelihood of reporting daily and COVID-19-related stressors. Because of the binary and count outcomes, these were fit as generalized multilevel model with a binomial or Poisson distribution, respectively (log link; SAS PROC GLIMMIX). Next, we examined the relations of COVID-19-related stressors and daily affect, after accounting for other types of daily stressors (SAS PROC MIXED). Finally, we included depressive disorder status (as described above) as a moderator to determine whether individuals with greater symptom severity were more reactive to COVID-19-related stressors compared to those without.

All models report unstandardized coefficients. For continuous variables, the coefficients indicate the amount of change in the outcome (e.g., negative affect) for a one-unit change in the predictor variable. For categorical variables (e.g., sex or stressor exposure), the coefficient represents the difference between groups at level 2 or types of days at level 1 (e.g., stressor vs. non-stressor days). Significant interaction effects were probed using simple slopes analysis to generate estimated slopes for each group. All models included the average total number of daily stressors and COVID-19-related stressors to control for differences between individuals on exposure to stress across the diary period. Models included student status, sex, race/ethnicity, and age (grand mean centered) as covariates based on our previous work examining affective reactivity (Stawski et al., 2008; Piazza et al., 2013). Given the design of this initial pilot study and the small analytical sample size, we had 80% power to detect a small-to-medium Cohen’s *f* (0.22). Data are reported as mean±SD and significance was set at *p*<0.05.

## Results

A total analytical sample of 58 adults (18 men; 22±3years) and 442days of data were available. Participants were racially diverse (38% white; 33% Asian; 8% Native American/Hawaiian; 8% Black; and 13% multi-racial) and 22% identified as Hispanic/Latinx (Table 1). Most participants were currently enrolled in classes (89%), while the remaining 11% were community-dwelling young adults (Table 1). Most participants were not living in on-campus university housing (84%). Exposure to daily stressors was reported on approximately half of the interview days (*n*_days_=220, 49.8%). Further, participants reported a COVID-19-related stressor on approximately one-third of interview days (*n*_days_=158, 35.8%), indicating overlap between other forms of daily stressors and COVID-19-specific stressors. Both types of stressors events were reported on 24% of days. The most common COVID-19-related stressor was distressing newscast exposure (*n*_days_=72; 16%). Of the total analytical sample, 20 (34%; *n*=14 female) reported a PHQ-9 score of ≥10 (18±2), indicating the presence of a depressive episode (Spitzer et al., 1999; Kroenke et al., 2001, 2010). Non-depressed adults reported a PHQ-9 of 4±3.0. The presence of a depressive episode did not predict the likelihood of any stressor exposure (*p*=0.26; OR=1.53, 95% CI: 0.726–3.235) or the total number of daily stressors (*p*=0.142; OR=1.31, 95% CI: 0.912–1.875). Similarly, a depressive episode was not a significant predictor of either the likelihood of stressor exposure (*p*=0.46; OR=1.52; 95% CI: 0.492–4.718) or the total number of COVID-19-related stressors (*p*=0.44; OR=1.317; 95% CI: 0.647–2.680).

**Table 1:** Descriptive characteristics.

	All participants	(−) Depressive episode	(+) Depressive episode
*n*	58	38	20
Age (years)	22±3	23±3	22±2
Sex (% women)	68	68	70
Student (%)	89	89	90
Hispanic/Latinx (%)	22	19	30
**Race (%)**			
White	38	46	25
Black or African American	8	11	5
Asian	33	27	45
Native Hawaiian or Other Pacific Islander	8	8	10
American Indian/Alaska Native	0	0	0
More than one race	13	8	15

*Data are mean±SD*.

**Figure 1:**
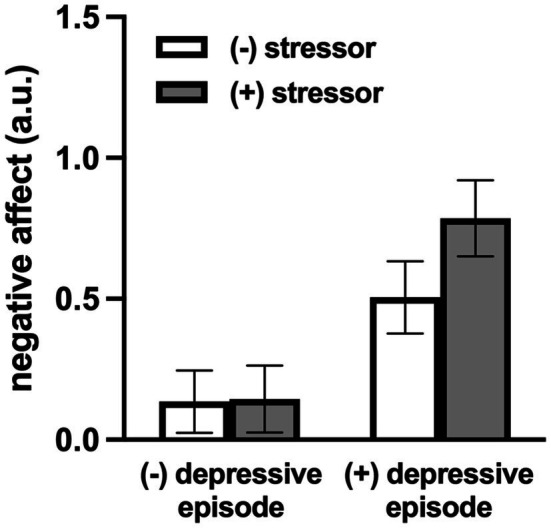
Model-estimated negative affect for individuals with and without a depressive episode, on stressor and non-stressor days. The magnitude of the increase in negative affect on days with an exposure to a daily stressor was greater in adults with a depressive episode (*p*=0.02). Data are mean±SE.

Days with a daily stressor exposure were characterized by greater negative affect and lower positive affect (Table 2; both *p*<0.01) compared to stressor-free days. Adults with a depressive episode reported greater negative affect on days with an exposure to any daily stressor (*b*=0.70, SE=0.077, *p*<0.01) compared to individuals without (*b*=0.33, SE=0.063, *p*<0.01). Further, the daily stress-related increase in negative affect was greater in adults with a depressive episode (Figure 1; *p*<0.01). Exposure to a daily stressor was associated with lower positive affect in both adults with (*b*=−0.42, SE=0.010, *p*<0.01) and without a depressive episode (*b*=−0.37, SE=0.08, *p*<0.01); this relation was not different between groups (*p*=0.68). Data were consistent when analyzing the total number of daily stressors.

**Table 2:** Daily stressor exposure, affective response, and depressive symptomology.

	Negative affect						Positive affect					
	Model 1			Model 2			Model 1			Model 2		
Variable	Estimate	SE	*p*	Estimate	SE	*p*	Estimate	SE	*p*	Estimate	SE	*p*
Intercept	0.044	0.12	0.728	0.141	0.13	0.267	2.376	0.24	<0.01	2.362	0.25	<0.01
Daily stressor (ref=“no stressor”)	0.478	0.05	<0.01	0.326	0.06	<0.01	−0.391	0.06	<0.01	−0.370	0.09	<0.01
Daily stressor×Depressive episode	–	–	–	0.378	0.10	<0.01	–	–	–	−0.052	0.13	0.686
Average number of daily stressors	0.039	0.21	0.855	0.112	0.21	0.592	−0.650	0.41	0.116	−0.660	0.41	0.112
Depressive episode (ref=“no”)	0.479	0.12	<0.01	0.276	0.13	0.033	−0.797	0.23	<0.01	−0.769	0.24	0.002
Student status (ref=“yes”)	−0.122	0.19	0.521	−0.153	0.19	0.415	0.842	0.37	0.028	0.846	0.37	0.028
Sex (ref=“male”)	0.251	0.12	0.042	0.231	0.12	0.058	−0.185	0.24	0.438	−0.183	0.24	0.446
Ethnicity (ref=“white”)	0.067	0.12	0.587	0.054	0.12	0.658	0.077	0.24	0.751	0.079	0.24	0.746
Age	−0.002	0.02	0.912	−0.002	0.02	0.908	−0.010	0.04	0.790	−0.010	0.04	0.791

*Multilevel models included a random intercept. Coefficients are unstandardized. For continuous variables, coefficients reflect the change in the outcome for a one unit change in the predictor. For categorical variables, coefficients reflect the difference between the reference group and the remaining category. Model 1 includes the main effects of all variables; Model 2 includes the interaction between a depressive episode and daily stressors to test for group differences*.

**Figure 2:**
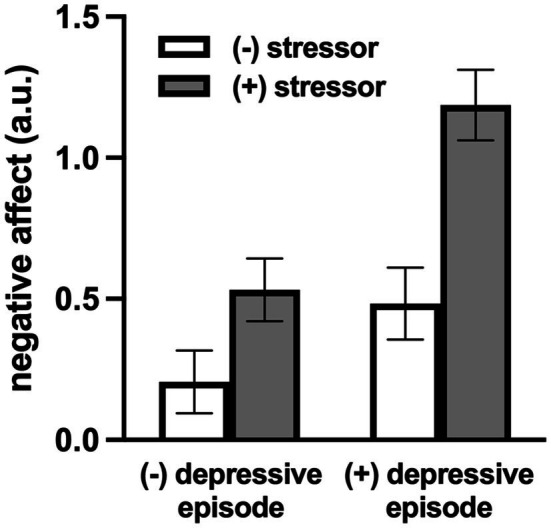
Model-estimated negative affect (NA) for individuals with and without a depressive episode, on COVID-19-specific stressor and non-COVID-19-specific-stressor days. NA was greater on days with an exposure to a COVID-19-specific daily stressor in adults with a depressive episode but not in those without. Data are mean±SE.

Exposure to COVID-19-specific daily stressors also appeared to be associated with negative affect, though this did not reach statistical significance (Table 3; *p*=0.062). COVID-19-related daily stressors were not related to positive affect (Table 3; *p*=0.764). Consistent with the aforementioned emotional response to non-COVID-19-related daily stress, adults with a depressive episode also had greater negative affect on days with exposure to any COVID-19-specific stressor (*b*=0.281, SE=0.093, *p*=0.003), whereas individuals without did not (*b*=0.009, SE=0.074, *p*=0.90), such that negative affective reactivity to daily COVID-19-related stressors was amplified in adults with a depressive episode (Figure 2; *p*=0.019). There were no associations between COVID-19-related stressor exposure and positive affect in either group, nor were there any differences in this response between groups (Table 3; *p*=0.673). Similar results were obtained when examining the total number of COVID-19-related stressors.

**Table 3:** COVID-19-specific daily stressor exposure, affective response, and depressive symptomology.

	Negative affect						Positive affect					
	Model 1			Model 2			Model 1			Model 2		
Variable	Estimate	SE	*p*	Estimate	SE	*p*	Estimate	SE	*p*	Estimate	SE	*p*
Intercept	0.015	0.13	0.907	0.063	0.13	0.621	2.410	0.25	<0.01	2.340	0.25	<0.01
COVID-19-related daily stressor (ref=“no stressor”)	0.112	0.06	0.062	0.009	0.07	0.900	−0.023	0.08	0.764	0.001	0.10	0.988
COVID-19-related daily stressor×Depressive episode	–	–	–	0.272	0.12	0.012	–	–	–	−0.065	0.15	0.673
Average number of COVID-19-related daily stressors	0.029	0.21	0.892	0.022	0.21	0.919	0.431	0.41	0.300	0.433	0.41	0.298
Other daily stressors	0.470	0.05	<0.01	0.465	0.05	<0.01	−0.390	0.06	<0.01	−0.389	0.06	<0.01
Average number of other daily stressors	−0.033	0.24	0.892	−0.008	0.24	0.974	−0.882	0.47	0.064	−0.888	0.47	0.063
Depressive episode (ref=“no”)	0.477	0.12	<0.01	0.370	0.12	0.004	−0.803	0.23	<0.01	−0.777	0.24	0.002
Student status (ref=“yes”)	−0.145	0.19	0.455	−0.153	0.19	0.422	0.775	0.38	0.046	0.777	0.38	0.046
Sex (ref=“male”)	0.232	0.12	0.068	0.229	0.12	0.066	−0.240	0.24	0.328	−0.239	0.24	0.330
Ethnicity (ref=“white”)	0.092	0.13	0.478	0.069	0.13	0.585	0.148	0.25	0.558	0.154	0.25	0.545
Age	−0.003	0.02	0.887	−0.001	0.02	0.954	−0.012	0.04	0.745	−0.012	0.04	0.738

*Multilevel models included a random intercept. Coefficients are unstandardized. For continuous variables, coefficients reflect the change in the outcome for a one unit change in the predictor. For categorical variables, coefficients reflect the difference between the reference group and the remaining category. Model 1 includes the main effects of all variables; Model 2 includes the interaction between depressive episode and COVID-19-related daily stressors to test for group differences*.

## Discussion

The primary novel finding of this small pilot study is that although neither exposure to, nor the total number of, COVID-19-related daily stressors were different between adults with and without a depressive disorder, both the likelihood of exposure and the cumulative total of exposures were associated with increased negative affective reactivity in adults with depression. The presence of a depressive disorder did not moderate positive affect. Taken together, these data suggest that daily stressors related to COVID-19 further worsen negative affect in adults with a depressive disorder. These findings add to the rapidly growing body of literature demonstrating that people with existing mental health illness are far more vulnerable to daily stress related to the COVID-19 pandemic.

Daily stressors, the common – albeit minor – naturalistic events or hassles that arise from day-to-day living, have both immediate and long-term consequences for psychological and physiological function (Almeida et al., 2009; Stawski et al., 2013; Greaney et al., 2019, 2020). In this regard, heightened negative affective reactivity to daily stressors is a powerful predictor of future depression (Charles et al., 2013), lending support to the concept that a sensitized emotional response to daily stress exposure may contribute to the susceptibility to mental health disorders (Caspi et al., 2010). Although, daily stress is uniquely predictive of emotional health and well-being (Twisk et al., 1999; Piazza et al., 2013; Stawski et al., 2013; Leger et al., 2018), chronic life stress necessarily influences the affective response to a daily stressor exposure (Stawski et al., 2008; Sliwinski et al., 2009). Interestingly, it is increasingly evident that the chronic stress of the ongoing global pandemic is also a significant source of novel daily stressors unique to the circumstances surrounding COVID-19 (Nelson and Bergeman, 2020; Klaiber et al., 2021; Sin et al., 2021; Zheng et al., 2021). Indeed, emerging data suggest that increased COVID-19-related daily stress (e.g., concern for one’s health, social isolation financial insecurity, etc.) is associated with increased emotional distress and depressive symptoms (Duan et al., 2020; Shanahan et al., 2020; Zheng et al., 2021). This effect appears even more pronounced in adults with pre-existing depressive symptomology (Zheng et al., 2021), suggesting an important link between COVID-19-related daily stress, affective responsiveness, and symptoms of depression.

To begin to probe these inter-relations in more detail, this small proof-of-concept study was designed to determine whether moderate-to-severe depressive symptomology modulates the affective response to COVID-19-related daily stressors. Broadly consistent with, and an important extension of, the available literature (Duan et al., 2020; Nelson and Bergeman, 2020; Shanahan et al., 2020; Zheng et al., 2021), the primary novel finding of the present study is that the presence of a depressive episode synergistically magnified the detrimental emotional consequences of a COVID-19-related daily stressor exposure (e.g., hearing distressing news reports). Based on the evidence that negative affective reactivity to daily stress is exacerbated by greater pandemic-related worry (Nelson and Bergeman, 2020), it was somewhat surprising that there was no effect of COVID-19-specific daily stress on negative affect in adults without depression. This is especially notable considering that we did, in fact, detect and confirm the expected association between “traditional” non-COVID-19-related daily stressors and negative affect (Bylsma et al., 2011; Booij et al., 2018). The reason(s) for this discrepancy is not entirely clear. Certainly, the lack of consensus in the literature on the specific daily stressors that constitute a COVID-19-related stress has not yet been definitively established, as this is a rapidly evolving area of research (Klaiber et al., 2021). Another possibility is that non-depressed adults may be better equipped to adapt and cope with pandemic-related stress (Hill et al., 2021; Killgore et al., 2020). Although, these possibilities clearly require more targeted investigation, based on the present findings, it appears that current symptoms of depression may be necessary to fully unmask the association between COVID-19-related daily stress and negative affective reactivity.

In contrast, depressive symptomology did not moderate the association between COVID-19-related daily stress and positive affect, though positive affect was reduced in adults with a depressive disorder compared to those without. This finding was somewhat surprising given that susceptibility to reduced positive emotions in the context daily stress appears to increase the risk for poor mental health outcomes, including anxiety and depressive disorders (Rackoff and Newman, 2020). Interestingly, the buffering capacity of positive affect to protect against heightened negative affective reactivity appears diminished when individuals are exposed to a COVID-19-specific stress (Nelson and Bergeman, 2020). Because adults with depression have less capacity for positive affect (Heller et al., 2009), this in turn may mechanistically contribute to amplified negative affective reactivity in the face of pandemic-related daily stress exposure. This causal mechanism of stress susceptibility merits additional study.

Age is a primary risk factor for severe illness and increased mortality risk stemming from COVID-19 infection (Zhou et al., 2020). However, studies have, perhaps surprisingly, consistently reported that older adults are less emotionally reactive to daily stress during the pandemic than young adults (Carstensen et al., 2020; Nelson and Bergeman, 2020; Novotny et al., 2020; Bruine de Bruin, 2021). As a result, there is an emerging body of research that has explored the potential factors contributing to increased mental distress in young adults during COVID-19 (Gao et al., 2020; Zheng et al., 2021). In this regard, and consistent with the data demonstrating that pre-menopausal women are more than twice as likely to develop depression and suffer greater depressive symptom severity (Kessler et al., 2003), the COVID-19 pandemic has had a more severe impact on the mental health of women (Luo et al., 2020; Novotny et al., 2020). Moreover, there is evidence that women are more emotionally reactive to daily stressor exposure than men, potentially contributing to increased risk of poor mental health outcomes (Almeida and Kessler, 1998). As such, we performed exploratory analyses to begin to examine whether greater COVID-19-related daily stress-induced increases in negative affect in young women mechanistically contribute to the aforementioned prevalence of depression during the pandemic in this cohort; however, these results did not reach statistical significance. However, given the small sample size of this initial study, future targeted investigations of the potential influence of sex on negative affective stress reactivity, in young adults both with and without depression, are warranted. Further, numerous stress-related behaviors (e.g., substance/tobacco use, sleep disturbances, unhealthy eating, etc.) have a bi-directional relation with depression (Musselman et al., 1998) and thus, in a feedforward manner may further exacerbate the severity of emotional responsiveness to stress exposure in adults with depressive symptoms. Consideration of these additional risk factors will be critical for future prospective studies designed to better understand the mechanistic underpinnings of daily stress reactivity and depression as the COVID-19 pandemic persists.

### Limitations

There are inherent limitations to this initial feasibility study that warrant consideration. First, the study included a relatively small sample size, which may limit data interpretation. However, sensitivity power analyses indicate a small-to-medium effect (Cohen’s *f*=0.22), providing preliminary support for the concept that depressive symptomology influences daily stress processes as they relate to the COVID-19 pandemic. Second, ~90% of the analytical sample were students. As such, the current findings may differ for young adults who are not currently enrolled in a higher education program, as well as for middle-aged and older adults. Third, it is not possible to discern the directionality of depressive symptomology and daily stress processes in the current study, owing to the lack of comparable data prior to the onset of the COVID-19 pandemic. Finally, because data were collected, while severe restrictions to in-person, laboratory-based human subjects research were in place, a formal clinical diagnosis of major depressive disorder was not feasible. Instead, participants were characterized based on depressive symptomology. Whether the magnitude of the increase in negative affect on days with an exposure to any daily stressor is related to the degree of depressive symptom severity should be considered in future investigations. Nevertheless, the results of this small, initial study provide novel insight to the role of depressive symptomology on daily stress processes during the COVID-19 pandemic in young adults, which likely has important implications for long-term health and well-being.

### Perspectives

Although, the specific COVID-19-related daily stressors have continued to evolve as the pandemic has persisted (e.g., a shift from quarantine and isolation-related stressors to those related to the lifting of emergency directives to vaccination-specific stressors and the emergence of SARS-CoV-2 variants), the current findings nevertheless add to the growing body of literature highlighting an amplification of the emotional response to stressor exposure during the pandemic, a link that may be driven by concurrent depression (Yao et al., 2020; Pan et al., 2021; Zheng et al., 2021). In addition to its inextricable link to the disease process of depression itself, stress system dysfunction is directly linked the initiation and progression of pathophysiological alterations that substantially increase cardiovascular disease risk and mortality (Dimsdale, 2008). In this regard, our laboratory recently demonstrated that daily stressor exposure was associated with greater impairments in vascular function in young otherwise healthy adults with depression (Greaney et al., 2019). Whether this link is also evident for COVID-19-related daily stress – and whether strategies to promote stress resistance and resilience during the pandemic, particularly in adults with depression – may mitigate or prevent untoward cardiovascular outcomes is an exciting avenue for future research.

## Data Availability Statement

The raw data supporting the conclusions of this article will be made available by the authors, without undue reservation.

## Ethics Statement

The studies involving human participants were reviewed and approved by the Institutional Review Board at the University of Texas at Arlington (2020-0912). The patients/participants provided their written informed consent to participate in this study.

## Author Contributions

JG, ES, DA, and JM contributed to conception and design of the study. JG, AD, and JM collected the data. JG, AD, JT, and JM analyzed the data and performed statistical analysis. JG, AD, JT, ES, DA, and JM interpreted the data. JG drafted the manuscript. All authors contributed to the article and approved the submitted version.

## Funding

This work was supported by National Institutes of Health (NIH) awards HL133414 (JG), MH123928 (JG), and AG062605 (JM and JT).

## Conflict of Interest

The authors declare that the research was conducted in the absence of any commercial or financial relationships that could be construed as a potential conflict of interest.

## Publisher’s Note

All claims expressed in this article are solely those of the authors and do not necessarily represent those of their affiliated organizations, or those of the publisher, the editors and the reviewers. Any product that may be evaluated in this article, or claim that may be made by its manufacturer, is not guaranteed or endorsed by the publisher.
